# Mineralocorticoid receptor signaling in GABAergic neurons regulates stress-induced cognitive flexibility

**DOI:** 10.1016/j.ynstr.2026.100845

**Published:** 2026-08-07

**Authors:** Huanqing Yang, Veronika Kovarova, Alena Godunova, Sowmya Narayan, Lotte van Doeselaar, Danusa Menegaz, Matthias Eder, Juan Pablo Lopez, Jan M. Deussing, Mathias V. Schmidt

**Affiliations:** a Research Group Neurobiology of Stress Resilience, Max Planck Institute of Psychiatry, Munich, Germany; b Research Group Translational Research in Psychiatry, Max Planck Institute of Psychiatry, Munich, Germany; c Core Unit Electrophysiology, Max Planck Institute of Psychiatry, Munich, Germany; d Department of Neuroscience, Karolinska Institute, Stockholm, Sweden; e Research Group Molecular Neurogenetics, Max Planck Institute of Psychiatry, Munich, Germany

**Keywords:** Mineralocorticoid receptor, GABAergic neurons, Stress resilience, Synaptic plasticity, Sex differences

## Abstract

The mineralocorticoid receptor (MR) plays a pivotal role in modulating the neuroendocrine stress response and cognitive function. While recent evidence highlights the importance of MRs in glutamatergic neurons in regulating anxiety-like behavior, the specific contribution of MRs within inhibitory networks remains incompletely understood. To address this gap, we generated a mouse model with targeted ablation of MR in forebrain GABAergic neurons (MR^Dlx^). Comprehensive behavioral profiling revealed a profound, state-dependent cognitive phenotype in male MR^Dlx^ mice. Under non-stressful baseline conditions, these mice exhibited impaired object recognition memory, while under aversive learning paradigms, such as the Morris water maze and fear conditioning, they displayed enhanced spatial and contextual memory. Furthermore, male MR^Dlx^ mice demonstrated significant behavioral lack of adaptation to 21 days of chronic social defeat stress (CSDS). Notably, these changes were highly sex-influenced, as female MR^Dlx^ mice did not exhibit the same baseline cognitive deficits or subsequent stress resistance. Mechanistically, *in vitro* hippocampal electrophysiological recordings from male mice showed that acute corticosterone (CORT) application, which typically suppresses long-term potentiation (LTP), failed to impair LTP in MR^Dlx^ slices, indicating a marked resistance to CORT-induced suppression. Together, our findings suggest that MRs in GABAergic neurons normally function as critical constraints on excitatory synaptic plasticity during high-stress states. Ablating this regulatory mechanism confers robust behavioral and synaptic stress resistance, underscoring that adaptive stress responses rely on a finely tuned, cell-type-specific balance of corticosteroid signaling within limbic microcircuits.

## 1 Introduction

The ability to mount an adaptive response to stressful stimuli is fundamental for survival and relies heavily on the finely tuned action of glucocorticoids. These hormones, specifically cortisol in humans and corticosterone (CORT) in rodents, are secreted from the adrenal gland after activation of the hypothalamic-pituitary-adrenal (HPA) axis (Herman et al., 2016). They operate primarily through two closely related receptors: the glucocorticoid receptor (GR) and the mineralocorticoid receptor (MR). While GRs are traditionally associated with the termination of the stress response following peak glucocorticoid levels, MRs bind CORT with a high affinity, maintaining basal tone and driving the initial, proactive appraisal of novel or stressful situations (de Kloet, 2022; De Kloet et al., 2005). In the brain, MRs are abundantly expressed throughout limbic structures, including the hippocampus, amygdala, and prefrontal cortex, and are critical modulators of emotional reactivity, neuroendocrine feedback, and cognitive function. Within these networks, MR signaling shapes how an organism processes environmental challenges and coordinates subsequent behavioral adaptations (Paul et al., 2022; Yang et al., 2023).

Historically, the functional significance of limbic MRs has been elucidated using ubiquitous or forebrain-wide deletion or overexpression models, such as the CaMKIIα-Cre system, or cell-type unspecific pharmacological manipulations. While these foundational studies established the indispensable role of MR in learning, memory, and affective behaviors (Berger et al., 2006a, Berger et al., 2006b; Brinks et al., 2009; Kanatsou et al., 2015a, 2015b; Lai et al., 2007; Rozeboom et al., 2007; Smythe et al., 1997), they inherently manipulated the receptor from diverse neuronal populations simultaneously. Cognitive flexibility and appropriate stress reactivity depend fundamentally on the dynamic interplay between excitatory and inhibitory neurotransmission. Because ubiquitous manipulation models affect MR from both glutamatergic projection neurons and GABAergic interneurons, the distinct, and potentially opposing, contributions of these specific microcircuits to the overall stress phenotype have remained largely obscured.

Recognizing this limitation, recent efforts have shifted toward dissecting MR function with strict cell-type-specific approaches. Single-cell transcriptomic profiling of the adult mouse hippocampus revealed that targeted deletion of MR in either glutamatergic or GABAergic neurons produces profoundly distinct, cell-type-specific transcriptional signatures at baseline and following acute stress (De Donno et al., 2026). Aligning with these molecular divergences, we recently demonstrated the discrete role of MRs located within excitatory glutamatergic neurons (Yang et al., 2026), which specifically in males resulted in an anxiety-like phenotype, accompanied by distinct transcriptomic alterations. This provided clear evidence that excitatory MRs are vital for mitigating anxiety-related behaviors in a sex-influenced manner. However, while the role of MR in glutamatergic neurons is now better understood, the precise physiological and behavioral functions of MRs within the GABAergic inhibitory network remain fundamentally uncharacterized.

To address this critical gap, the present study investigates the selective contribution of GABAergic MRs to stress reactivity and cognitive behavior. By employing the Dlx-Cre mouse line, we generated a model with targeted MR ablation restricted to forebrain GABAergic neurons (MR^Dlx^). We hypothesized that the loss of MR within limbic inhibitory networks would yield a distinct and partially opposed behavioral and physiological profile compared to our previous findings of MR deletion in glutamatergic neurons, with a predominant effect under stressful conditions. Indeed, our results reveal that, in contrast to the phenotype observed in glutamatergic MR knockouts, the deletion of GABAergic MRs uniquely enhances cognitive performance under stressful conditions, highlighting a profound functional divergence of MR signaling based on neuronal subtype.

## 2 Materials and methods

### 2.1 Animals

The creation of MR-floxed mice was previously described (Berger et al., 2006a, Berger et al., 2006b). To conditionally knock out MR in forebrain GABAergic neurons, MR^lox/lox^ mice, where exon 3 is flanked by loxP sites, were crossed with Dlx-Cre animals (Monory et al., 2006, Fig. 1A). Cre activity begins during development, starting at embryonic day E10. The resulting offspring with a deletion of MR in GABAergic neurons (MR^lox/lox^-Dlx-Cre, referred to as MR^Dlx^) and their control MR^lox/lox^ littermates (referred to as Ctrl) were used (De Donno et al., 2026). All mouse lines used were on a mixed 129S2/Sv×C57BL/6 genetic background backcrossed to C57BL/6NRj. Experimenters were always blind to genotypes. Genotyping was performed by PCR and primer sequences are available on request.

**Fig. 1:**
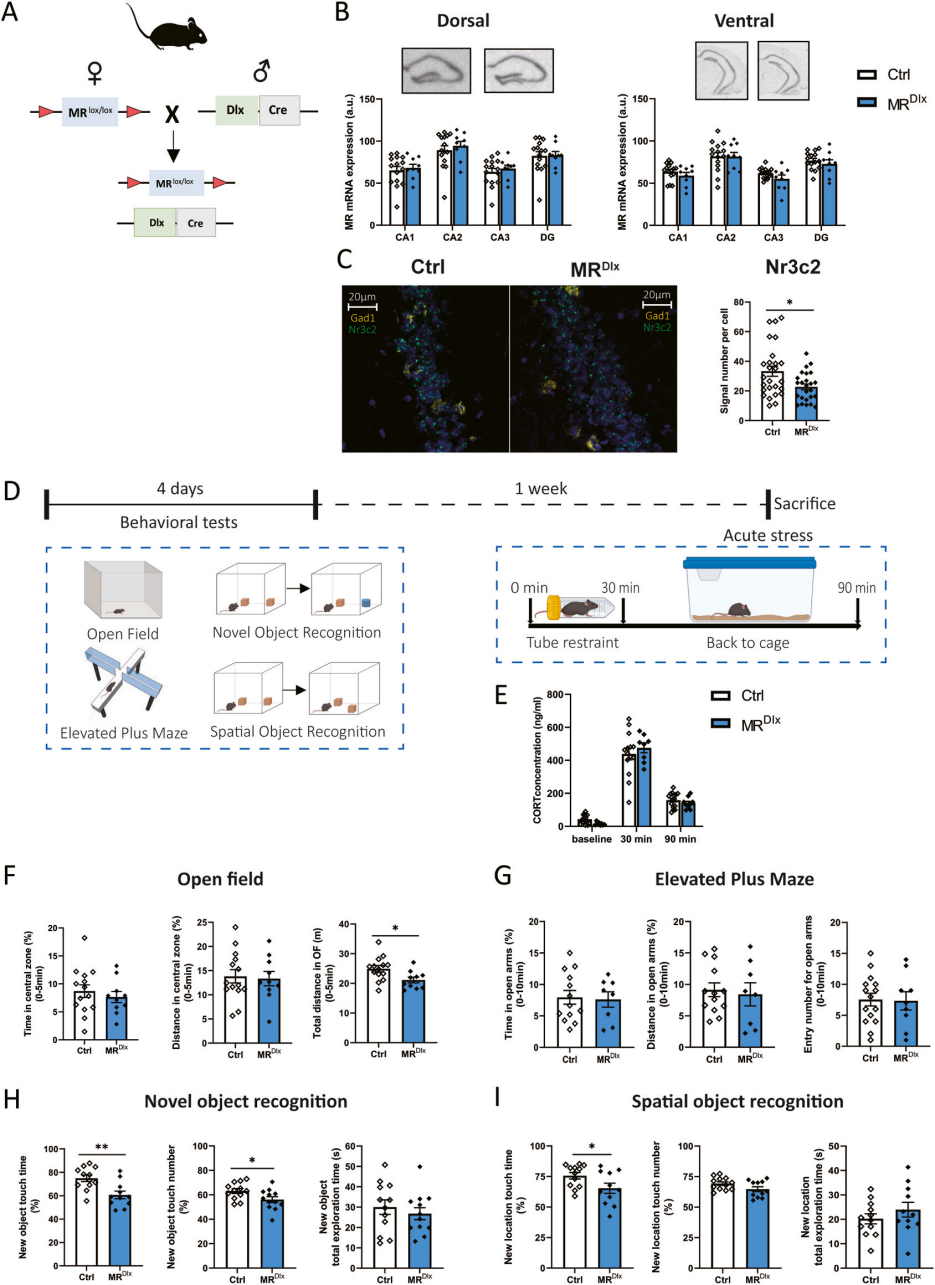
**Breeding and validation of MR^Dlx^ mice, baseline behavioral experimental measurements**. **(A)** Breeding scheme of MR^Dlx^ mice. **(B)** MR mRNA levels were not significantly reduced in the dorsal or hippocampus when assessed with in-situ hybridization. WT: n = 15, MR^DLX^: n = 9 **(C)** RNAscope analysis confirmed a cell type-specific reduction of MR mRNA (Nr3c2) expression (puncta per GABA-positive cell) in GABAergic neurons. N = 3 mice per genotype; individual data points represent individual GABA-positive cells. **(D)** Schematic of the experimental time line **(E)** CORT levels were significantly increased in both groups of mice following acute restraint, with no effect of genotype. In the OF test **(F)** no effect of genotype was detected for anxiety-related measures (percentage time and distance in the center zone), but a moderate decrease in total distance traveled. In the EPM test **(G)**, lack of MR in GABAergic neurons had no significant effect on the behavioral performance of mice. The cognitive function of MR^Dlx^ and control mice was tested in the NOR test **(H)** and SOR test **(I)**. Percentage of novel object compared to total object interaction time and number was significantly reduced in the NOR, without affecting total exploration time. Similarly, percentage of spatial object compared to total object interaction time was significantly reduced in the SOR, without affecting the percentage of number of object interactions and total object interaction time. (E)-(I): WT: n = 15, MR^DLX^: n = 10 * p < 0.05, **p < 0.01.

All studies were conducted in conformity with the European Communities' Council Directive 2010/63/EU, as well as the Guidelines for the Care and Use of Laboratory Animals of the Government of Upper Bavaria, Germany. The mice used in the experiments were young adults ranged in age from 8 to 20 weeks and both sexes were included in separate cohorts. Unless otherwise specified, during the experiment mice were housed individually in cages (IVC; 30 cm × 16 cm × 16 cm; 501 cm^2^) with adequate bedding and nesting material and wooden tunnels for environmental enrichment 1 week prior to behavioral testing or hormonal assessment. Animals were kept in a standard laboratory environment with *ad libitum* food (Altromin 1318, Altromin GmbH, Germany) and water, a central airflow system (Tecniplast, IVC Green Line-GM500), and maintained in a 12:12 h light-dark cycle (lights on at 07.00 a.m.) at a constant temperature of 23 ± 2°C and 55% humidity.

### 2.2 Stress paradigms

Restraint stress was utilized for acute stress (Paré and Glavin, 1986). Mice were restrained into 50 ml falcon tubes with a hole in the top and the lid, respectively, for 30 min. At the end of 30 min, tail blood was collected from the mice (Fluttert et al., 2000), and blood samples were collected in 1.5 ml EDTA-coated microcentrifuge tubes (Kabe Labortechnik, Germany) for CORT level testing. The mice were then put back in their cages for 60 min, and a second sample of tail blood was collected at 90 min after the start of the restraint stress.

The chronic social defeat stress (CSDS) paradigm is commonly used to induce anxiety- and depression-related behaviors in mice and was performed as previously described (Bordes et al., 2023; Golden et al., 2011; van Doeselaar et al., 2021; Wagner et al., 2011). For males, mice in the CSDS group were directly placed in the home cage of a new dominant CD1 male every day. The animals were separated by a perforated divider following an aggressive fight and 2-3 attacks or after a maximum of 5 min, thereby avoiding any injuries on the test animals. For female mice, in order to provoke a male CD1 resident attack, mice were first covered with urine, which was collected from 15 to 20 C57Bl/6n male mice every 3–4 days, pooled, stored at 4°C, brought to room temperature before use (van Doeselaar et al., 2021). A brush was used to apply the urine to several parts of the body (head, back, tail and especially at the vaginal orifice). Defeat times were randomly assigned between 1 p.m. and 4 p.m. in the afternoon. Active and successful defeat was visually confirmed during the encounters for both males and females. Animals in the control group were kept in their home cages for 21 days under general animal facility husbandry, without exposure to aggression. During the experiment, all mice were weighed weekly, and the fur condition was scored daily (Mineur et al., 2003).

### 2.3 Behavioral experiments

All behavioral tests were performed in a dedicated test room, adjacent to the animal housing room. To prevent any potential behavioral alterations due to circadian rhythmic changes in CORT levels, tests took place between 8 a.m. and 1 p.m. The interval between behavioral tests was at least 24 h to minimize carry-over effects. The automated video tracking system ANY-maze (ANY-maze 6.18; Stoelting Co) was used to record, track, and assess the tests. If a manual assessment was required, it was performed by skilled scientists who were blinded to the experimental conditions.

### 2.4 Assessment of home cage behavior

Home cage locomotion test was conducted on animals in order to evaluate their basal activity. The mice were individually placed in cages equipped with an infrared mouse motion detector/data logger (Mouse-E-Motion; Infra-E-Motion, Hamburg, Germany). Measurements were performed for 72 consecutive hours, during which time mice had free access to food and water. The data are displayed as total number of seconds in which any movement occurred in 30-min intervals.

### 2.5 Open field test

The OF test is commonly utilized in studies of exploratory behavior and anxiety-like behaviors. As previously described (Schmidt et al., 2007; Sterlemann et al., 2008), the open field used for the test is made of grey polyvinyl chloride plastic material with a size of 50 cm × 50 cm x 50 cm. The test lasted for a total of 15 min in low light conditions (about 15 lux). During the test, the mice were placed in a corner of the open field and were allowed to explore the arena freely. The results were evaluated by dividing the total period into three 5-min segments, to analyze the distance the mice moved in the central area, the corners, and the entire open field, and time the mice spent in those areas.

### 2.6 Elevated plus maze test

The EPM test is used to assess anxiety-like behavior. As previously described (Schmidt et al., 2007; Sterlemann et al., 2008), the elevated maze apparatus used in the test was made of grey polyvinyl chloride plastic material with two opposing open arms (30 cm × 5 cm × 0.5 cm) and two opposing closed arms (30 cm × 5 cm × 15 cm), connected into a cross shape by a middle platform (5 cm × 5 cm) with a height of 50 cm. The test was conducted under low light settings (approximately 10 lux in the closed arms and 20 lux in the open arms) for a total of 10 min. During the test, the mice were placed on the middle platform area facing the closed arm, and allowed to explore the arms freely. The results were evaluated analyzing the percentage of the distance the mice moved in the open arms, the percentage of time spent in the open arms, and the number of times the mice entered the open arms.

### 2.7 Novel object recognition and Spatial Object Recognition tests

Compared with familiar objects, rodents naturally tend to spend more time exploring unfamiliar one (Ennaceur, 2010). The Novel Object Recognition (NOR) test assesses recognition memory and learning, while the Spatial Object Recognition (SOR) test assesses spatial memory. The objects used in the experiment were made by 13 Lego® bricks. For the NOR, two different objects variable in shape and color yet created to attain a consistent volume were used, while for the SOR two identical objects were used. To eliminate the mice's unfamiliarity, the experimental arena uses a large open home cage (425 × 276 × 153 mm), with bedding on the bottom of the cage. At the beginning of the experiment, the mice were given a 15-min familiarization period, and two same objects were placed 5 cm from the rear of the arena, and the mice were then placed in the arena away from the objects and allowed to explore freely. Following the familiarization period, the mice were returned to their home cages for 20 min before a 5-min testing period. In the NOR, one of the two identical objects was replaced by a different object in the arena, the mouse was placed in the same position of the arena as before. For the SOR, one of the objects was moved to a novel location, while the other one remained in the same location. Once the mouse touched an object with its nose, front paws, or whiskers, it was considered an interaction. Videos were manually scored using ANY-maze software, analyzing the ratio of time, and number of times the mice explored objects.

### 2.8 Morris water maze test

The MWM test is a cognitive task designed to evaluate spatial learning and memory in mice. In our experiment, the MWM test was carried out in a white circular pool with a diameter of 150 cm and a depth of 41 cm. The pool was placed in the center of the room with visual cues in the room on the surrounding walls. The pool was filled to a depth of 33 cm, and the water temperature was maintained at 22-23°C throughout the test. The room was illuminated with indirect light and the light on the water surface reaches 11.5 lux. The escape platform is a cylinder made of 8 cm × 8 cm transparent acrylic plexiglass, which was placed in the center of the north-east (NE) quadrant, 35 cm away from the wall, and submerged 1 cm below the water surface.

First, the mice were allowed to adapt to the pool and water for one day, after which they underwent a 5-day training period to find the platform. Each animal performed 4 trials per day. The starting points for each of the four trials were randomly distributed along the south-west (SW) area of the tank's perimeter and were each used for a different starting position. The mouse was gently lowered into the water facing the wall and was allowed to explore the pool for a maximum of 90 s. If the platform was located, it was allowed to stay on the platform for 10 s. If the mouse did not find the platform within 90 s, it was guided onto the platform without direct handling and remained there for 10 s before being taken from the pool. Inter-trial intervals were 15 min, which the mice spent in their home cage under a heating lamp to avoid hypothermia. On day 6, mice were subjected to a 60-s probe trial, for which the platform was removed. Time to reach the platform and time and distance traveled in each of the quadrants was recorded.

### 2.9 Fear conditioning

Fear conditioning and retrieval sessions were conducted using a Ugo Basile apparatus (Italy), with freezing behavior automatically recorded and analyzed via ANY-maze 6.18 software (Stoelting). The protocol was adapted from (Dias and Ressler, 2014). For fear acquisition (Day 1), mice were placed into a square conditioning chamber scented with 80% ethanol. Following a 1-min habituation period, the mice received five pairings of a conditioned stimulus (CS; 9 kHz, 80 dB tone for 29.5 s) and an unconditioned stimulus (US; 0.6 mA foot shock for 0.5 s) that co-terminated with the CS. Each pairing was separated by a 5-min inter-trial interval. The percentage of time spent freezing during the tone presentations was quantified. Twenty-four hours post-training (Day 2), contextual memory was assessed. Mice were re-exposed to the original conditioning chamber (identical shape and 80% ethanol odor) for 5 min in the absence of any auditory or aversive stimuli. Total freezing time during this 5-min period was measured. To assess cued fear memory, mice were placed on the subsequent day (Day 3) in a novel environment, consisting of a triangular chamber scented with 1% acetic acid. After a 1-min habituation period, mice were exposed to 10 presentations of the original CS tone (9 kHz, 80 dB for 30 s) separated by a 1.5-min inter-trial interval, with no foot shock administered. The percentage of time spent freezing in response to the tones was recorded.

### 2.10 Electrophysiology

Mice at the age of 8-12 weeks were anesthetized with isoflurane and decapitated. The brain was rapidly removed from the cranial cavity and, using a vibratome (HM650V, Thermo Scientific), 350 μm-thick horizontal slices containing the ventral hippocampus were cut in an ice-cold carbogen gas (95% O_2_/5% CO_2_)-saturated solution consisting of (in mM): 87 NaCl, 2.5 KCl, 25 NaHCO_3_, 1.25 NaH_2_PO_4_, 0.5 CaCl_2_, 7 MgCl_2_, 10 glucose, and 75 sucrose. Slices were incubated in carbogenated physiological saline for 30 min at 34°C and, afterwards, for at least 30 min at room temperature (23-25°C). The physiological saline contained (in mM): 125 NaCl, 2.5 KCl, 25 NaHCO_3_, 1.25 NaH_2_PO_4_, 2 CaCl_2_, 1 MgCl_2_, and 10 glucose. All measurements were conducted at room temperature. Slices assigned to the CORT condition (Fkbp5Nex CORT and Fkbp5lox/lox CORT) were stored for 1 h in carbogenated physiological saline, containing 1 μM CORT (Sigma-Aldrich Corticosterone, product nr. 27840, dissolved in 0.01% EtOH; Merck KGaA, Darmstadt, DE).

For long-term potentiation (LTP) and paired-pulse facilitation (PPF) experiments, slices were superfused with carbogenated physiological saline (4-5 ml/min flow rate). Field excitatory postsynaptic potentials (fEPSPs) at CA3-CA1 synapses were evoked by square-pulse electrical stimuli (50 μs pulse width) delivered via a bipolar tungsten electrode (50 μm pole diameter, ∼0.5 MΩ nominal impedance) to the Schaffer collateral-commissural pathway. fEPSPs were recorded using glass microelectrodes (filled with physiological saline, ∼1 MΩ open-tip resistance) that were placed into the CA1 stratum radiatum. The intensity of voltage stimulation was adjusted in a manner to produce a fEPSP of ∼50% of the amplitude at which a population spike appeared. Recording data were low-pass filtered at 1 kHz and digitized at 5 kHz. Before and after LTP induction, a single stimulation pulse was delivered every 15 s to the neural tissue. LTP was induced by high-frequency stimulation (HFS, 100 Hz for 1 s). Paired-pulse ratio was calculated by dividing the slope of fEPSP2 by the slope of fEPSP1.

### 2.11 Corticosterone measurements

All blood samples were collected and stored on ice, then centrifuged at 8000 rpm for 15 min at 4°C. Afterwards, 10 μl of plasma was transferred to newly labeled microcentrifuge tubes and stored in a −20°C freezer. CORT levels were assessed by Double Antibody 125I Radioimmunoassay Kit (MP Biomedicals Inc., Eschwege, Germany; sensitivity 12.5 ng/ml) or Enzyme-linked Immunosorbent Assay (ELISA) kit (RE52211, TECAN, IBL Hamburg, Germany) following the manufacturer's instructions.

### 2.12 Tissue collection and processing

Animals were killed by decapitation under isoflurane anesthesia. The basal trunk blood was collected, the adrenal glands were removed and weighed, and brains were quickly frozen and stored at −80°C before being sectioned.

### 2.13 *In-situ* hybridization (ISH)

*In-situ* hybridization was performed as previously described (Schmidt et al., 2007). In short, brain slices at 20 μm were fixed in 4% (v/v) PFA, dehydrated and exposed to a 35S-UTP-labeled antisense MR riboprobe. Following overnight hybridization at 55°C the sections were washed, dried and exposed to Kodak Biomax MR films. Autoradiographs were digitized and optical density for expression analysis was measured using the NIH ImageJ software.

### 2.14 RNAscope analysis and cell counting

The RNAscope Fluorescent Multiplex Kit (Item 320850, Advanced Cell Diagnostics, Newark, CA, USA) was used on 20 μm brain sections following manufacturer's instructions. Specific RNAscope probes targeting mouse Nr3c2 (mm-Nr3c2-C2) and Gad1 (mm-Gad1-C3) were used. All images were acquired using a Zeiss laser scanning confocal microscope and Zen software, using a 40× objective (n = 3 animals per marker and condition). All images were collected under the same imaging conditions. Each image was acquired in a 1.0 μm z-stack. The mRNA in the cells was quantified and analyzed using ImageJ and QuPath 0.3.2 software.

### 2.15 Statistical analysis

The IBM SPSS Statistics 22 (IBM SPSS Statistics, IBM) software was used to analyze the data, and GraphPad Prism 8.0 was used to create graphics (GraphPad Software). An independent Student's t-test was used to compare two groups. The data were tested for normality using the Shapiro-Wilk test and QQ plot. If the data were not normally distributed the nonparametric Mann–Whitney *U* test was applied. Data from more than two groups were analyzed with a two-way ANOVA model followed by Tukey post hoc analysis to determine statistical significance between groups. Time series data were analyzed by a two-way repeated measures ANOVA followed by Tukey post hoc analysis. If behavioral data were not normally distributed, a log transformation was utilized before analysis. The ANOVA significance levels were set at p < 0.05 for main effects and p < 0.1 for interaction effects. The significance threshold was set at p < 0.05 for each post hoc test. Values outside two standard deviations were considered outliers and excluded from the analysis. Data are visualized as mean ± standard error of the mean (SEM).

## 3 Results

Loss of MR in GABAergic neurons in male mice negatively affects cognitive function under non-stress conditions.

To generate mice with a specific deletion of the mineralocorticoid receptor (MR) in GABAergic neurons, we crossed female MR^lox/lox^ mice with male Dlx-Cre mice (MR^Dlx^) (Fig. 1A). Overall MR expression analyzed with in situ hybridization (ISH) did not reveal a significant reduction of MR mRNA in the CA1, CA2, CA3, and dentate gyrus (DG) regions (Fig. 1B), likely attributable to the relatively low abundance of GABAergic interneurons within the broader hippocampal cell population. Cell-type specific quantitative RNAscope analysis confirmed that MR expression was significantly reduced specifically within the GABAergic neurons of MR^Dlx^ mice compared to controls (t (49) = 2.651, p = 0.011), validating the successful generation of the desired genotype (Fig. 1C) and confirming the previous validation of this line (De Donno et al., 2026). The remaining signal measured in MR^Dlx^ GABAergic cells is likely due to the detection of unfunctional truncated MR mRNA transcripts that are still transcribed following successful Cre-recombination.

Following genotype confirmation, male MR^Dlx^ and control mice (aged 12–20 weeks) underwent a battery of behavioral tests to assess baseline anxiety-like behavior and cognitive function (Fig. 1D). After the completion of behavioral testing, the mice were given a one-week resting period to ensure a return to an unstressed baseline before assessing hypothalamic-pituitary-adrenal (HPA) axis reactivity. Following an acute restraint stress paradigm, CORT levels significantly increased in both MR^Dlx^ and control mice at 30 min and decreased 1 h after returning to their home cages. This response was comparable between genotypes, with a significant main effect of time (F (2,63) = 191.0, p < 0.001), but no significant main effect of genotype (F (1,63) = 0.0305, p = 0.8619), indicating comparable stress induced CORT secretion and subsequent negative feedback regulation in both groups (Fig. 1E). Baseline anxiety-like behavior was evaluated using the OF and EPM tests. In the OF test, although MR^Dlx^ mice traveled a shorter total distance compared to control mice (t (22) = 2.509, p = 0.020), there were no significant differences in the percentage of time spent in the central zone (t (22) = 0.675, p = 0.507) or the percentage of distance traveled in the center (t (22) = 0.235, p = 0.816; Fig. 1F). Similarly, the EPM test revealed comparable anxiety levels between the genotypes; MR^Dlx^ mice exhibited no significant differences from controls regarding the time spent in the open arms (t (22) = 0.2014, p = 0.751), the distance traveled in the open arms (t (22) = 0.3672, p = 0.368), or the number of entries into the open arms (t (22) = 0.1139, p = 0.696; Fig. 1G).

Cognitive function was assessed using the NOR and SOR tests. Both assays indicated an impairment in the MR^Dlx^ mice, as control mice spent a significantly higher percentage of object exploration time exploring the novel objects (NOR: t (21) = 3.348, p = 0.003; SOR: t (21) = 2.213, p = 0.047; Fig. 1H–I). Moreover, control mice demonstrated a significantly higher percentage of frequency of interactions with the novel object during the NOR test (t (22) = 2.259, p = 0.034; Fig. 1F), though this interaction percentage did not reach statistical significance in the SOR test (t (21) = 1.835, p = 0.081). Importantly, these differences were not driven by baseline exploration deficits, as there was no significant difference between the two groups in the total time spent exploring both objects combined (NOR: t (22) = 0.690, p = 0.497; SOR: t (21) = 1.042, p = 0.309; Fig. 1H–I). Together, these data suggest that the ablation of MR in GABAergic neurons impairs baseline object recognition memory performance in male mice without significantly altering baseline anxiety levels.

### 3.1 Improved spatial memory of male mice lacking MR in GABAergic neurons in stressful learning tasks

**Fig. 2:**
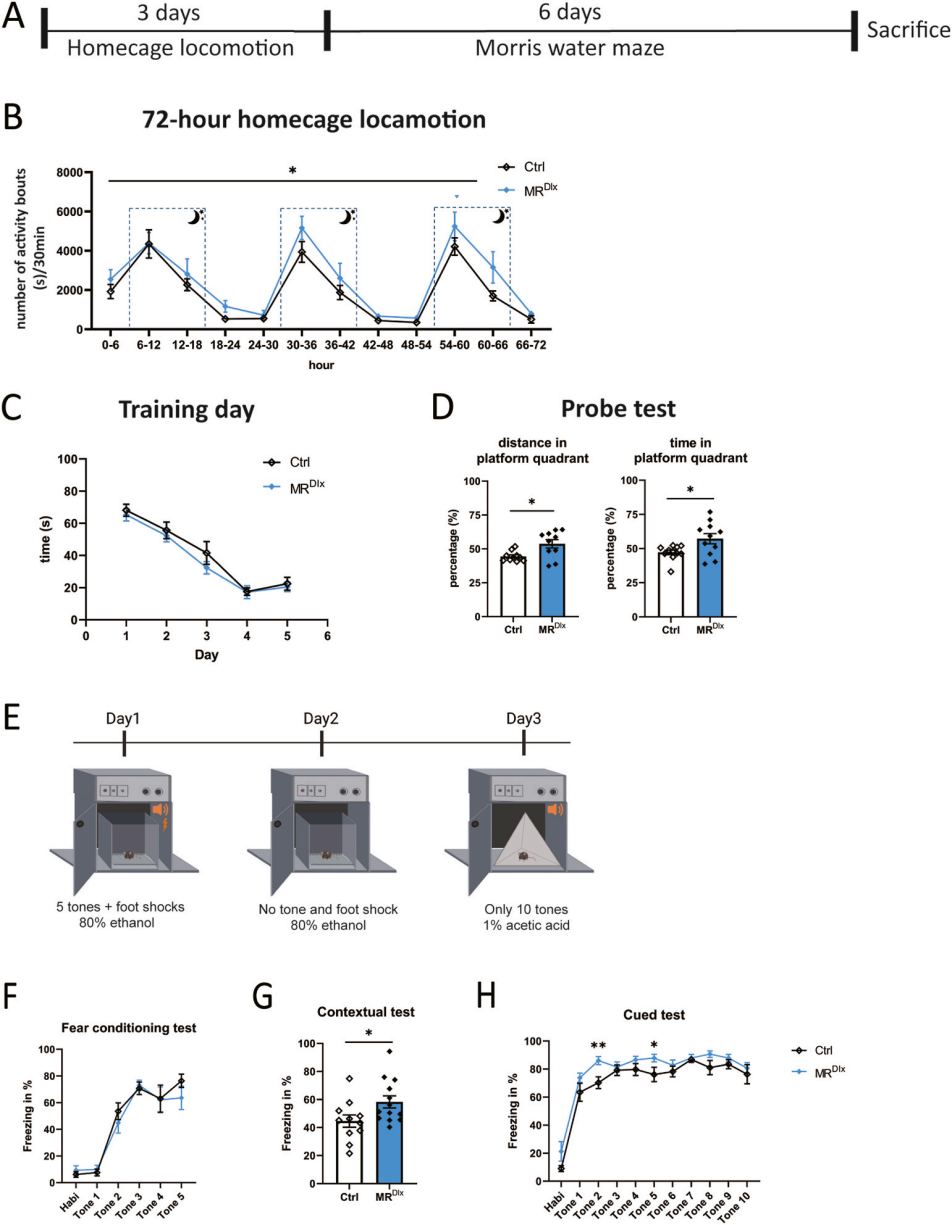
**Experimental design and results for assessing home cage activity levels and spatial learning in male mice. (A)** Experimental design. **(B)** Home cage locomotor activity over 72 h. Both groups showed circadian activity patterns, with significant main effects of genotype and time but no genotype × time interaction. WT: n = 6, MR^DLX^: n = 7 **(C)** Spatial memory acquisition in the MWM was unaffected by genotype over the course of the 5 training days. **(D)** Loss of MR in GABAergic neurons improved spatial memory recall in the MWM, with a higher percentage of distanced traveled and time spent in the previous platform location quadrant. WT: n = 12, MR^DLX^: n = 12 **(E)** Fear conditioning test procedure. **(F)** Acquisition of conditioned fear was unaffected by the loss of MR in GABAergic neurons. **(G)** MR^DLX^ mice showed a significantly higher freezing response to the conditioned context. **(H)** MR^DLX^ mice displayed a higher percentage of freezing to the conditioned stimulus. WT: n = 12, MR^DLX^: n = 14 * Genotype effects with * p < 0.05, **p < 0.01; # Quadrant effect with ####p < 0.0001.

Given the baseline cognitive alterations observed, we next evaluated continuous home cage locomotion and performance in a stressful learning paradigm using the MWM in a separate cohort of mice (Fig. 2A). Continuous homecage monitoring revealed that both MR^Dlx^ and control mice exhibited typical circadian activity rhythms. Because locomotor activity counts were right-skewed, the data were log-transformed before statistical analysis. Two-way repeated-measures ANOVA of log-transformed locomotor activity revealed a significant main effect of genotype, indicating overall increased home cage activity in MR^Dlx^ mice compared to controls (F (1,11) = 5.541, p = 0.038). There was also a significant main effect of time (F (11,121) = 32.599, p < 0.0001), whereas the genotype × time interaction was not significant (F (11,121) = 0.469, p = 0.919; Fig. 2B).

In the MWM test, there were no apparent genotype differences during the acquisition phase. Both groups demonstrated a comparable, gradual decrease in escape latency to find the hidden platform, indicating that spatial learning acquisition was not impaired in MR^Dlx^ mice (F (1, 109) = 1.693, p = 0.196; Fig. 2C). However, during the subsequent probe trial, while quadrant occupancy analysis revealed a significant main effect of quadrant (F (3, 88) = 80.35, p < 0.0001), MR^Dlx^ mice exhibited a markedly altered performance. Specifically, MR^Dlx^ mice spent a significantly higher percentage of time in the NE target quadrant than control mice during the probe trial (t (20) = 2.453, p = 0.024) and traveled a significantly higher percentage of their total swimming distance in this quadrant (t (18) = 2.784, p = 0.012) compared with the control group (Fig. 2D). Based on these findings, MR^Dlx^ mice display a more persistent and rigid memory performance following a stressful learning task.

Because the MWM procedure is inherently aversive, we hypothesized that the altered cognitive performance of MR^Dlx^ mice might be driven by the stressful nature of the paradigm. To further investigate learning and memory under fear-inducing conditions, a third cohort of mice was subjected to fear conditioning (FC) (Fig. 2E). During the acquisition phase on the first day, both genotypes successfully acquired the conditioned fear response across the five tone-shock pairings, with no significant difference in performance (F (1, 104) = 0.161, p = 0.689; Fig. 2F). In the contextual fear retrieval test conducted on the second day, however, MR^Dlx^ mice exhibited a significantly higher percentage of freezing time in the conditioning chamber compared to the control group (t (22) = 2.206, p = 0.038; Fig. 2G). During the cued fear retrieval test on the third day, genotype differences were not uniformly observed across all conditioned stimulus presentations. Instead, MR^Dlx^ mice showed selectively increased freezing during the 2nd (t (24) = 3.164, p = 0.004) and 5th (t (24) = 2.104, p = 0.046) tone presentations, whereas freezing levels during the other tones did not differ significantly between genotypes (Fig. 2H). Together, these results align with the MWM findings, reinforcing the conclusion that the ablation of MR in GABAergic neurons leads to a more inflexible spatial and contextual memory when male mice are exposed to a stressful learning environment.

### 3.2 Male MR^Dlx^ mice are partially resistant to the consequences of chronic social stress

**Fig. 3:**
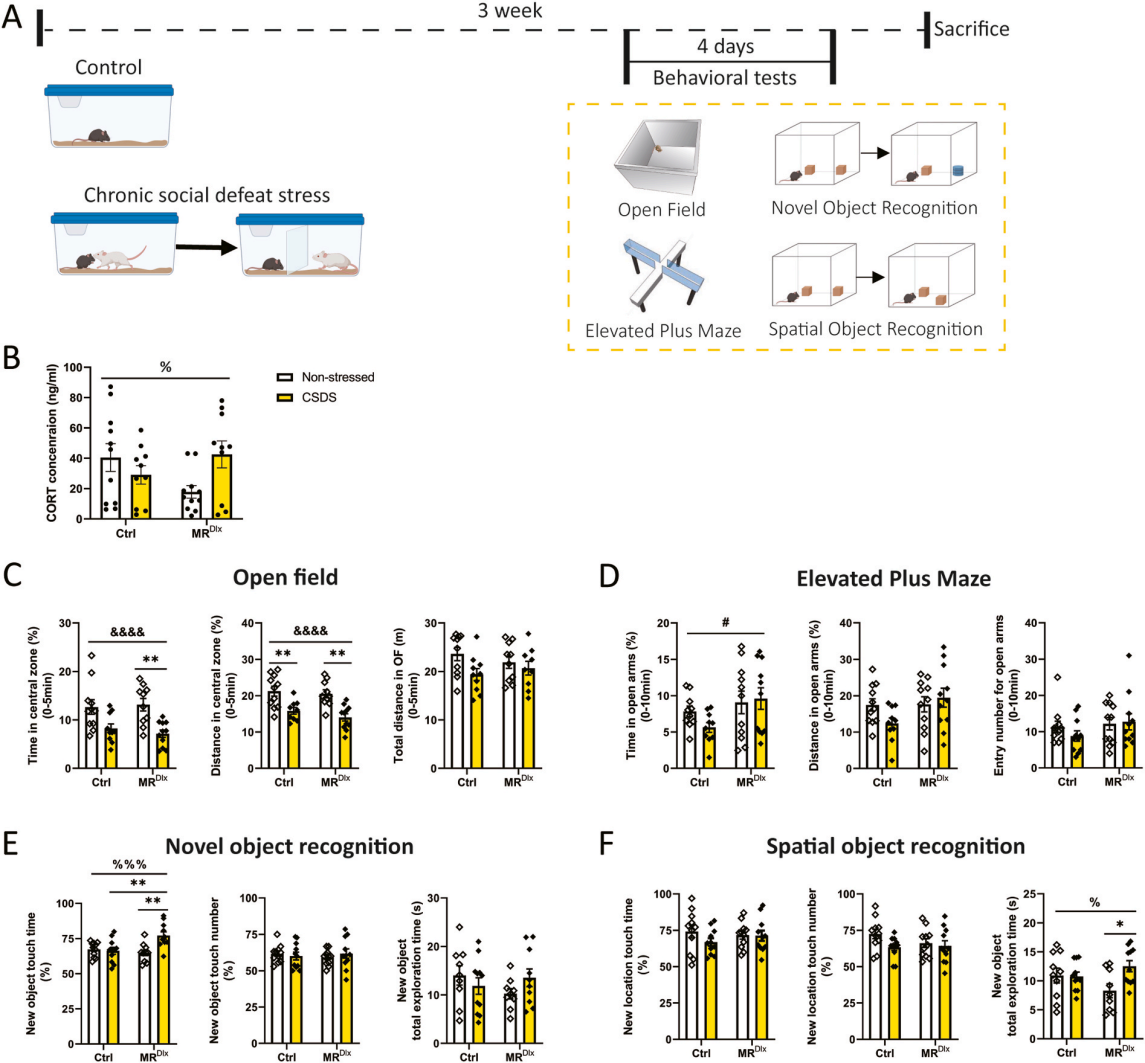
**Behavioral studies on male mice under chronic social defeat stress. (A)** Experimental timeline. The duration of the CSDS was 21 days, with behavioral tests carried out during the third week of CSDS with one behavioral test per day. N = 11 for all groups. **(B)** There was an interaction between genotype and stress in CORT level, with MR^Dlx^ mice displaying lower CORT at baseline, but not following CSDS. In the OF test **(C)** after CSDS, both MR^Dlx^ mice and Ctrl mice exhibited decreased activity duration and distance in the center of the OF arena, with no significant differences between the two genotypes. In the EPM test **(D)** after CSDS, both MR^Dlx^ mice and Ctrl mice exhibited decreased activity duration and distance in the open arms, with no significant difference between the genotypes. In the NOR test **(E)**, a genotype × stress interaction was observed, with MR^DLX^ mice showing a significantly higher percentage of novel object interaction time following CSDS. Percentage object interaction number and total exploration time were not affected by CSDS or genotype. In the SOR test **(F)**, genotype or CSDS had no significant effect on cognitive measures, but total object exploration time was significantly increased specifically in stressed MR^DLX^ mice. *p < 0.05, **p < 0.01, ***p < 0.001. **#** ANOVA genotype effect with ^#^p < 0.05, ^##^p < 0.01, ^###^p < 0.001. **&** ANOVA treatment (CSDS) effect with ^&^p < 0.05, ^&&^p < 0.01, ^&&&^p < 0.001. **%** ANOVA interaction between genotype and treatment with **^%^**p < 0.05, **^%%%^**p < 0.001.

Having established that short-term, acute stressors enhance cognitive performance in MR^Dlx^ mice, we next investigated whether these mice exhibit altered emotional and cognitive responses following prolonged stress exposure. To this end, a separate cohort of mice was subjected to a CSDS paradigm for 21 days, with a battery of behavioral tests conducted between days 15 and 18 of the stress protocol (Fig. 3A). Following the completion of the experiments, plasma CORT levels were measured. While there were no significant main effects of genotype (F (1, 38) = 0.383, p = 0.539) or stress condition (F (1, 38) = 0.815, p = 0.337), a significant genotype-by-stress interaction was observed (F (1, 38) = 5.995, p = 0.019; Fig. 3B). This interaction reflected an opposite direction of genotype differences between non-stressed and CSDS conditions; however, post-hoc comparisons did not detect significant genotype differences within either condition.

In the OF test, mice exposed to CSDS spent significantly less time (F (1, 38) = 19.84, p < 0.0001) and traveled less distance (F (1, 37) = 28.07, p < 0.0001) in the central zone of the arena compared to the non-stressed groups, reflecting expected stress-induced avoidance behavior. However, there were no significant main effects of genotype for the percentage of time spent in the central zone (F (1, 38) = 0.068, p = 0.796), the percentage of distance traveled in the center (F (1, 37) = 1.248, p = 0.271), or the total distance traveled (F (1, 35) = 0.023, p = 0.879; Fig. 3C).

In the EPM test, control mice subjected to CSDS exhibited a reduction in the percentage of time spent and distance traveled in the open arms compared to their non-stressed counterparts. Conversely, this stress-induced anxiogenic effect was absent in the MR^Dlx^ group. This divergence was supported by a significant genotype-by-stress interaction for the percentage of time spent in the open arms (F (1, 38) = 4.490, p = 0.041; Fig. 3D).

During the NOR test, MR^Dlx^ mice exposed to CSDS exhibited a higher percentage of time exploring the novel object compared to CSDS-exposed control mice, highlighted by a significant interaction between chronic stress and genotype (F (1, 39) = 8.890, p = 0.047). However, no significant main effects of stress or genotype were observed for the frequency of novel object exploration (stress: F (1, 40) = 0.046, p = 0.832; genotype: F (1, 40) = 0.029, p = 0.867), nor for the total time spent exploring the new object (stress: F (1, 35) = 0.106, p = 0.746; genotype: F (1, 35) = 0.370, p = 0.547; Fig. 3E). In the SOR test, a significant genotype-by-stress interaction was detected for total object exploration time (F (1, 37) = 4.417, p = 0.042). Nevertheless, there were no significant main effects of stress or genotype regarding the percentage of time spent exploring objects in novel locations (stress: F (1, 40) = 1.259, p = 0.269; genotype: F (1, 40) = 0.075, p = 0.786) or the frequency of exploring objects in novel locations (stress: F (1, 40) = 3.268, p = 0.078; genotype: F (1, 40) = 0.790, p = 0.379; Fig. 3F).

Taken together, the repeated emergence of significant genotype-by-stress interactions across selected assays suggests that the ablation of MR in GABAergic neurons confers a degree of task-specific behavioral and cognitive resistance to the effects of chronic social stress.

### 3.3 Basal and chronic stress-induced behavioral differences in female MR^Dlx^ are less pronounced compared to males

**Fig. 4:**
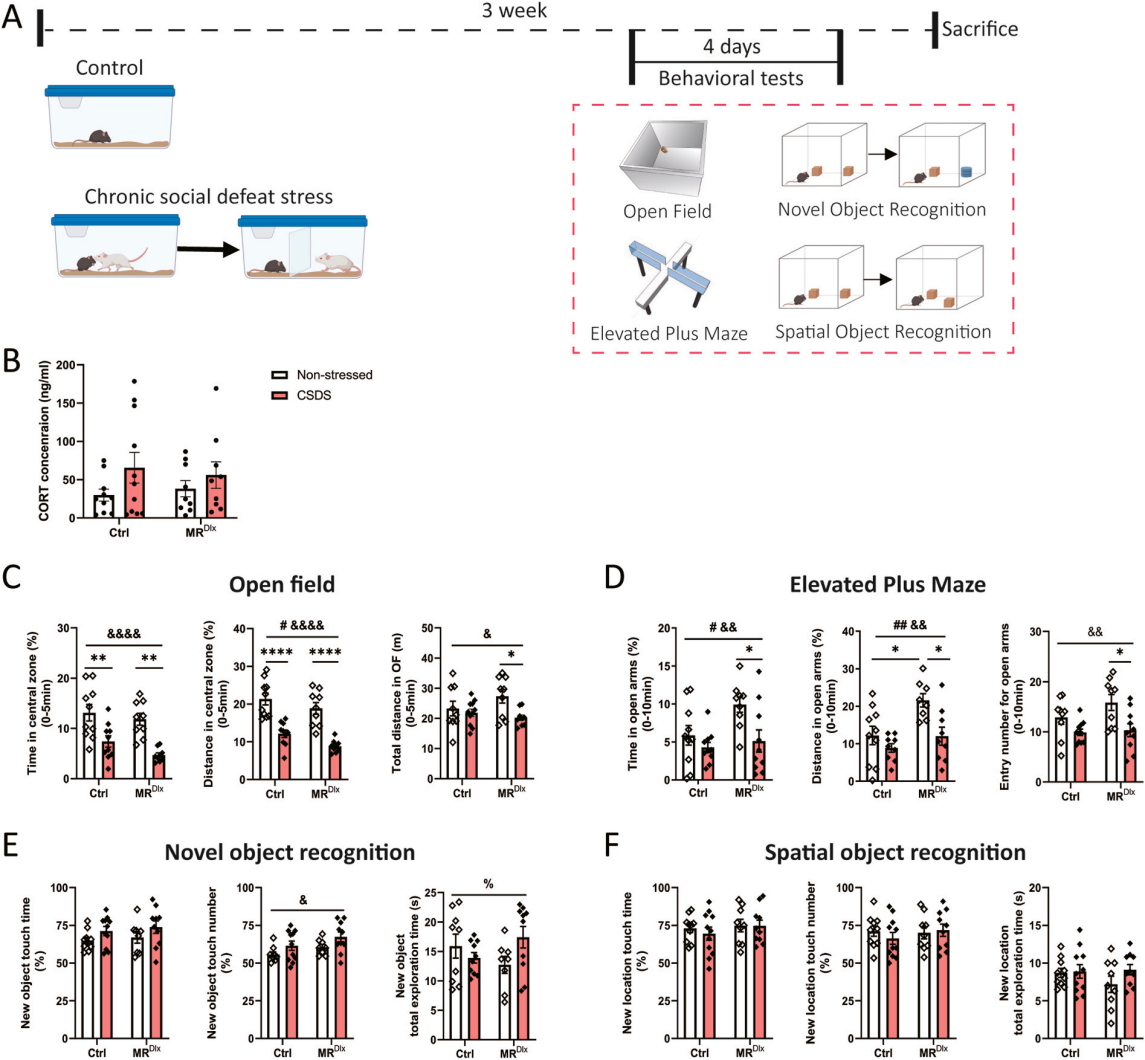
**Behavioral studies on female MR^Dlx^ mice under chronic social defeat stress. (A)** Experimental time line. The duration of the CSDS was 21 days, with behavioral tests carried out during the third week of CSDS with one behavioral test per day. N = 9-11 per group. **(B)** CORT levels of female mice did not differ significantly between the groups. **(C)** After CSDS, both MR^Dlx^ mice and Ctrl mice exhibited decreased activity duration and distance in the center of the open field arena, with no significant differences between the genotypes. **(D)** MR^Dlx^ mice were less anxious than Ctrl under non-stressed conditions, but there was no significant difference between genotypes after CSDS, where both genotypes displayed increased anxiety. **(E)** In the NOR test, CSDS females showed a higher percentage number of novel object interactions, with no effect of genotype or interaction, and percentage novel object investigation time was also not affected. **(F)** In the SOR test, no genotype or CSDS dependent effects were detected. *p < 0.05, **p < 0.01, ***p < 0.001. **#** ANOVA genotype effect with ^#^p < 0.05, ^##^p < 0.01, ^###^p < 0.001. **&** ANOVA treatment (CSDS) effect with ^&^p < 0.05, ^&&^p < 0.01, ^&&&^p < 0.001. **%** ANOVA interaction between genotype and treatment with ^%^p < 0.05.

It is well established that physiological and behavioral stress responses are highly sex-influenced. Therefore, to determine whether the effects of GABAergic MR ablation differ by sex, we also evaluated a cohort of female MR^Dlx^ and control mice. Utilizing a modified chronic social stress paradigm (van Doeselaar et al., 2020), female mice were subjected to 21 days of CSDS, with behavioral testing conducted between days 15 and 18 (Fig. 4A). Following the stress paradigm, plasma CORT levels were measured. No significant main effects were found for genotype (F (1, 35) = 0.001, p = 0.971) or stress treatment (F (1, 35) = 3.040, p = 0.090; Fig. 4B).

In the OF test, no baseline variations were observed between the genotypes in the non-stressed groups. However, across all animals, exposure to CSDS significantly reduced both the time spent (F (1, 34) = 28.09, p < 0.0001) and the distance traveled (F (1, 34) = 59.28, p < 0.0001) in the central area of the arena. In females, CSDS-exposed mice exhibited significantly less total movement in the open field compared to their non-stressed counterparts, as indicated by a significant main effect of stress in the two-way ANOVA (F (1, 34) = 5.747, p = 0.022; Fig. 4C).

The EPM results revealed that mice subjected to CSDS exhibited a lower percentage of time spent (F (1, 35) = 7.637, p = 0.009) and distance traveled (F (1, 32) = 9.701, p = 0.004) in the open arms compared to the non-stressed groups. Interestingly, under baseline (non-stressed) conditions, MR^Dlx^ females spent a significantly higher percentage of time (t (16) = 2.964, p = 0.009) and traveled a greater percentage of distance (t (17) = 2.429, p = 0.026) in the open arms than control females. However, two-way ANOVA revealed no significant genotype-by-stress interactions for the percentage of time in the open arms (F (1, 35) = 1.925, p = 0.174), the percentage of distance in the open arms (F (1, 32) = 2.296, p = 0.139), or the total distance traveled (F (1, 35) = 1.112, p = 0.299; Fig. 4D).

In the NOR test (Fig. 4E), CSDS-exposed mice demonstrated a trend toward increased frequency of novel object exploration compared to non-stressed mice (F (1, 34) = 3.871, p = 0.057), but this did not reach statistical significance. CSDS exposure did not significantly affect the percentage of time exploring the novel objects compared to non-stressed mice (F (1, 35) = 0.4470, p = 0.497). Furthermore, an interaction effect was reported for the overall object exploration time (F (1, 33) = 4.321, p = 0.046). During the SOR test (Fig. 4F), although both the percentage of time spent and the frequency of exploring objects in the novel location were above chance levels (50%), no statistical differences were observed between the non-stressed and CSDS groups for any of the measured indicators (percentage of time exploring the novel position: F (1, 36) = 0.234, p = 0.631; frequency of exploring the novel position: F (1, 36) = 0.154, p = 0.697; total time spent exploring the novel position: F (1, 36) = 1.632, p = 0.209). Likewise, genotype did not significantly affect these parameters (percentage of time exploring the novel position: F (1, 36) = 0.840, p = 0.365; frequency of exploring the novel position: F (1, 36) = 0.318, p = 0.576; total time spent exploring the novel position: F (1, 36) = 0.592, p = 0.446).

Similar to our previous findings in the MR-Nex mouse model (Yang et al., 2026), these data indicate that the emotional behavior and cognitive performance of female MR^Dlx^ mice differ from those observed in male MR^Dlx^ mice mainly in the strength and consistency of the phenotype, with genotype-dependent effects being less pronounced in females.

### 3.4 LTP in mice lacking MR in GABAergic neurons is resistant to CORT-induced suppression

**Fig. 5:**
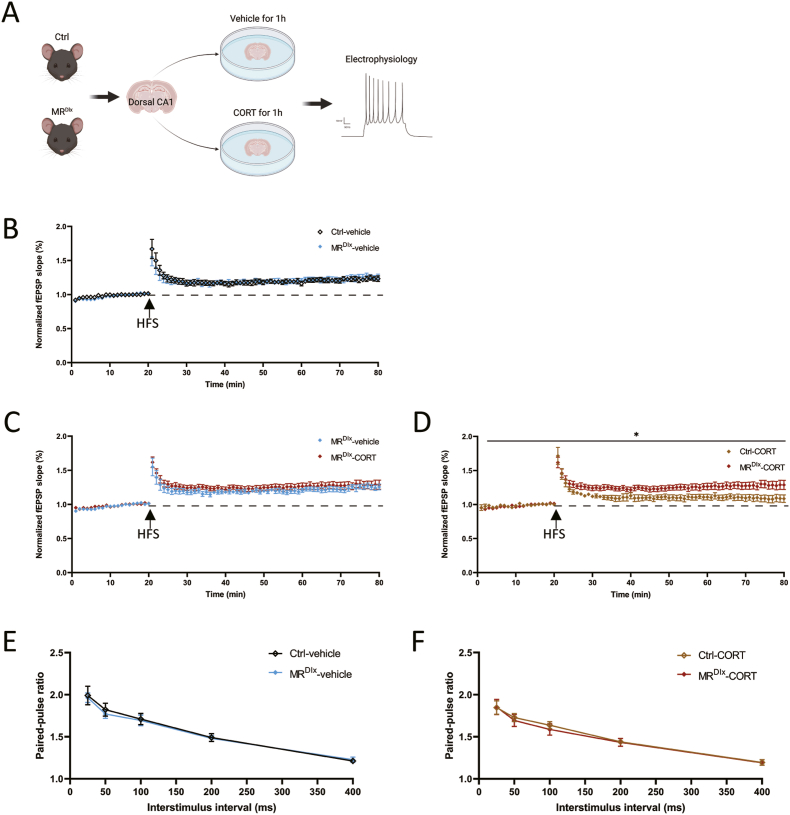
**Functional alterations of hippocampal LTP in MR^Dlx^ mice. (A)** Experimental design. **(B-D)** Average LTP fEPSP slope was reduced in Ctrl male mice but was unaffected in MR^Dlx^ male mice following CORT (1 μM) treatment. **(E-F)** The paired-pulse ratio of MR^Dlx^ mice and Ctrl mice was not significantly different in vehicle group and CORT group. N = 4 mice/8-10 slices per group. *p < 0.05.

Based on our behavioral findings, the cognitive performance of male MR^Dlx^ mice was enhanced following acute stress, but this cognitive enhancement was not sustained under chronic stress conditions. To investigate the synaptic mechanisms underlying this acute stress-induced cognitive resilience, we conducted *in vitro* electrophysiological recordings on acute hippocampal slices from male MR^Dlx^ and control mice. Slices were incubated either in a vehicle solution (0.01% EtOH) or treated with 1 μM CORT to simulate an acute stress exposure (Fig. 5A). The vehicle group consisted of slices from control mice (n = 5 mice, 8 slices) and MR^Dlx^ mice (n = 6 mice, 10 slices), while the CORT group included slices from control mice (n = 5 mice, 8 slices) and MR^Dlx^ mice (n = 5 mice, 8 slices).

Interestingly, under basal (vehicle) conditions, post hoc simple-effects comparison following two-way ANOVA revealed there was no significant difference in the average fEPSP slope during LTP maintenance between MR^Dlx^ and control mice (p = 0.9878; Fig. 5B–D). However, following CORT application, LTP in control mice was markedly suppressed, whereas LTP in MR^Dlx^ mice remained largely unaffected (two-way ANOVA genotype by treatment interaction (F (1, 30) = 3.831, p = 0.0597; Fig. 5C and D)). Consequently, in the CORT-treated group, post hoc simple-effects comparison following two-way ANOVA showed that LTP in control mice was significantly lower than LTP in MR^Dlx^ mice (p = 0.026).

Furthermore, to assess potential presynaptic alterations, we measured the paired-pulse ratio (PPR). There were no significant differences in the PPR between MR^Dlx^ and control mice in either the vehicle-treated (F (1, 80) = 0.219, p = 0.641) or the CORT-treated groups (F (1, 70) = 0.230, p = 0.633; Fig. 5E and F). Together, these electrophysiological data suggest that, in male mice, the deletion of MR in GABAergic neurons prevents the typical CORT-induced suppression of hippocampal LTP, providing a plausible cellular mechanism for the altered cognitive performance observed in male MR^Dlx^ mice following short-term stress.

### 3.5 Single-cell RNA sequencing identifies MR-specific target genes in GABAergic neurons

**Fig. 6:**
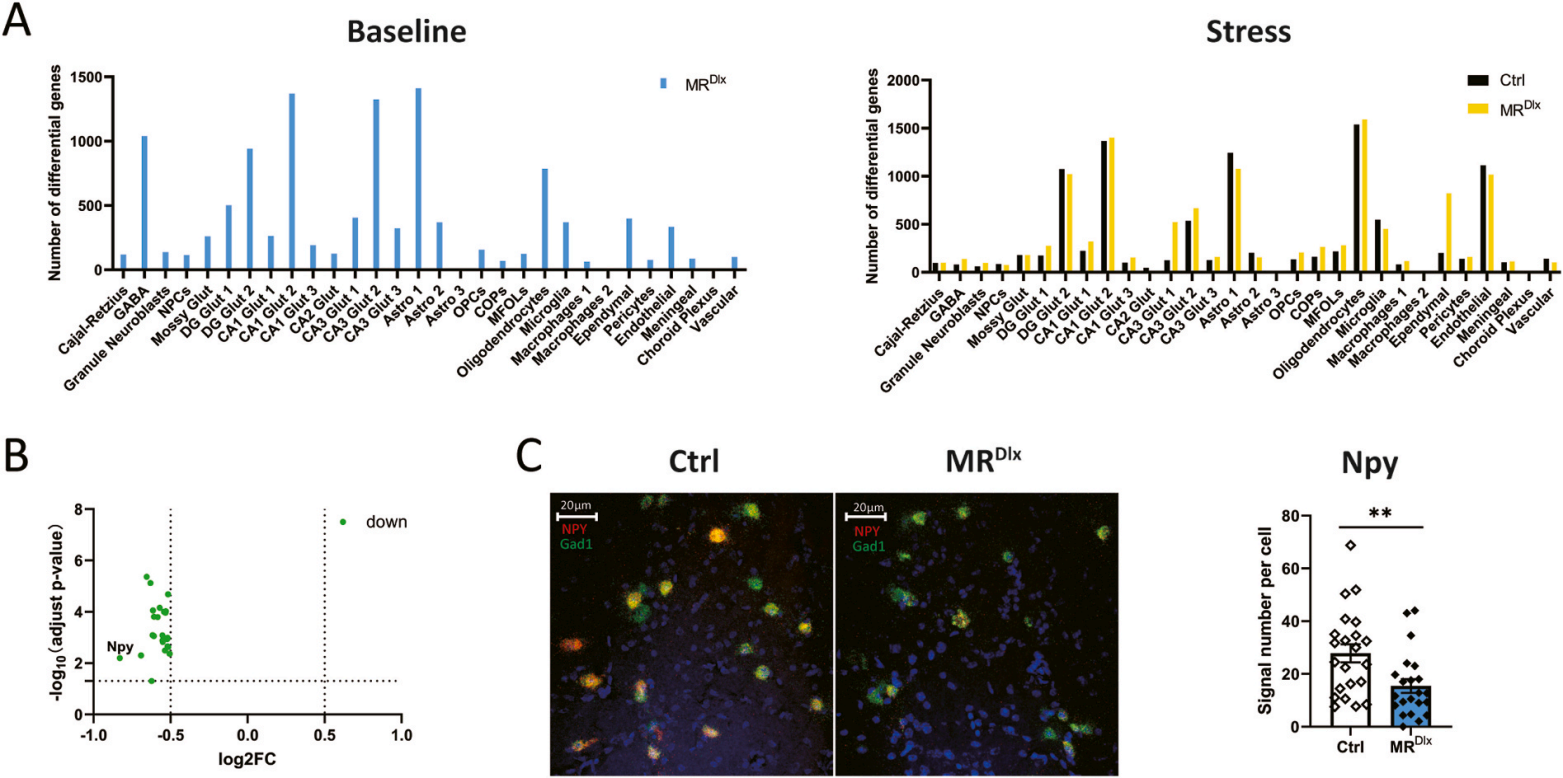
**Single-cell RNA sequencing data of MR^DLX^ animals**. **(A)** Histogram showing the number of differentially expressed genes at baseline in MR^Dlx^ animals and the number of differentially expressed genes in MR^Dlx^ animals following stress induction in the respective cell types. **(B)** Volcano plot showing the log2FC pattern of differentially expressed genes. **(C)** Validation of NPY downregulation in MR^DLX^ mice (*t*_*(42)*_ = *2.866, p = 0.006*). N = 3 mice per group; individual data points represent NPY positive cells. ∗Significant group difference at ∗∗p < 0.01. Ctrl, control; FC, fold change.

To investigate potential downstream mediators that could be driving the functional and behavioral differences observed following MR deletion in GABAergic neurons, we utilized a previously published single-cell RNA sequencing data set from the ventral hippocampus of wild-type and MR^DLX^ mice under control or acute stress conditions (De Donno et al., 2026). As shown in Fig. 6A, there were numerous differentially expressed genes in the different cell types under baseline conditions in MR^Dlx^ mice, while wild-type and MR^Dlx^ mice showed largely overlapping responses to acute stress. Within GABAergic neurons, there were 1040 differentially expressed genes at baseline. A total of 400 genes were selected based on a fold change of >|0.5|, and significant differences between Ctrl and MR^Dlx^ at the baseline, or between the non-stressed group and stress group within each genotype. To ensure sufficient expression levels, a threshold of >0.5 was set. Ultimately, 24 genes were discovered that exhibited differential expression exclusively in GABAergic neurons and not in other cells (Supplemental Table 1). Notably, NPY was identified among these genes (Fig. 6B). NPY is a neuropeptide that is highly expressed in certain subsets of GABAergic neurons in the brain and plays a crucial role in regulating stress responses and emotional behavior (Kupcova et al., 2022; Lach and de Lima, 2013). Subsequently, differential NPY expression was validated using RNAScope; consistent with the single cell RNA sequencing data the expression of NPY in MR^Dlx^ mice was significantly reduced compared to controls (t (42) = 2.866,p = 0.006).

## 4 Discussion

The mineralocorticoid receptor (MR) is a critical regulator of the neuroendocrine stress response and cognitive function, yet its specific role within distinct neuronal subpopulations has remained elusive. In the present study, we utilized the Dlx-Cre mouse line to selectively ablate MRs in forebrain GABAergic neurons. Our findings reveal a profound and highly specific behavioral and electrophysiological phenotype that fundamentally contrasts with previous ubiquitous or glutamatergic-specific MR manipulation models. We demonstrated that the loss of GABAergic MRs impairs baseline cognitive function in male mice and reduces spatial and contextual memory flexibility under stressful learning conditions. Furthermore, male MR^Dlx^ mice exhibited behavioral resistance and a lack of adaptation to CSDS, an effect that was highly sex-influenced, as female MR^Dlx^ mice displayed distinct and less pronounced stress-induced phenotypes. Mechanistically, *in vitro* electrophysiological recordings revealed that LTP in the hippocampus of MR^Dlx^ mice is remarkably resistant to CORT-induced suppression, providing a cellular basis for the observed stress-induced cognitive phenotype.

A striking finding of this study is the divergence in cognitive performance depending on the arousal or stress state of the animal. Under non-stressful, baseline conditions, MR^Dlx^ mice exhibited deficits in object recognition memory (NOR and SOR). However, when subjected to inherently aversive or highly stressful learning paradigms, such as the Morris water maze and fear conditioning, MR^Dlx^ mice displayed apparent superior memory retention, caused by a more rigid and less flexible memory recall. These findings are in contrast to unselective MR manipulations, where several investigations have demonstrated that non-contextual delivery of stress or use of the MR antagonist spironolactone prior to contextual fear conditioning can lessen contextual fear by interfering with memory formation via non-genomic effects (Sajadi et al., 2006; Zhou et al., 2010, 2011). Further, forebrain MR-deficient female mice were unable to distinguish between cued and contextual fears as accurately as male mice in a fear conditioning memory test (ter Horst et al., 2012). These findings underscore that in GABAergic neurons MR function is distinct and differs from that in other neuronal cell types. The stress-dependent cognitive rigidity observed in male MR^Dlx^ mice is strongly supported by our electrophysiological data. Typically, elevated CORT levels associated with acute, severe stress act to suppress hippocampal LTP, thereby constraining memory consolidation (Diamond and Rose, 1994; Pavlides et al., 1993). In our study, CORT application successfully suppressed LTP in control slices, mirroring this well-documented stress-induced impairment. In stark contrast, LTP in MR^Dlx^ slices were resistant to CORT-induced suppression. This suggests that under normal physiological conditions, MRs in GABAergic interneurons may play a gating role on plasticity at excitatory synapses during periods of high stress or elevated CORT, enabling stress-adapted behavior. By removing this brake, the hippocampal circuitry in MR^Dlx^ mice remains unaffected even in highly aversive environments, thereby reducing cognitive flexibility. A further interesting aspect is the observed suppressed locomotor activity in a novel environment, but increased locomotion in the home cage of MR^Dlx^ males. This finding points to the possibility that these measures reflect distinct behavioral processes, where locomotion in arousal states are differentially regulated to locomotion in home environments. This underscores the importance of including home cage behavior in addition to arousal-based test situations.

The profound functional dichotomy of neuronal MRs becomes particularly evident when contrasting the current findings with our recent investigation of MR deletion in excitatory networks. Using a glutamatergic-specific knockout model (MR^Nex^), we previously demonstrated that the loss of MRs in excitatory neurons predominantly drives an anxiety-like phenotype and alters neuroendocrine reactivity (Yang et al., 2026). In stark contrast, male MR^Dlx^ mice exhibited no such baseline anxiety alterations, but rather a unique cognitive resistance under stress. This behavioral divergence aligns seamlessly with recent single-cell transcriptomic profiling of the adult mouse hippocampus, which revealed that targeted deletion of MR in either glutamatergic or GABAergic neurons yields fundamentally distinct, cell-type-specific transcriptional signatures following acute stress (De Donno et al., 2026). Using this data set, we uncovered a unique set of MR-specific target genes in GABAergic neurons and validated the differential expression of NPY as a promising candidate to mediate such effects. Together, these complementary findings suggest that glutamatergic MRs primarily contribute to maintain basal emotional tone and mitigating anxiety, whereas GABAergic MRs act as facilitators of cognitive flexibility and memory consolidation during aversive states. The ultimate behavioral adaptation to stress, therefore, relies on the homeostatic balance of MR signaling in these interconnected and functionally opposing neuronal subtypes.

Importantly, this cell-type-specific effect extends beyond acute challenges, as evidenced by the resistance of male MR^Dlx^ mice to chronic social stress. Prolonged stress exposure typically induces a number of behavioral adaptations, predominantly increased anxiety-like behavior and cognitive blunting (Hartmann, 2025; Scott et al., 2025; Wagner et al., 2012). In our study, while control mice exhibited the expected stress-induced behavioral differences, male MR^Dlx^ mice were largely resistant against these outcomes, demonstrating significant genotype-by-stress interactions across different behavioral domains. This suggests that the ablation of MRs in inhibitory networks not only prevents the acute CORT-induced suppression of synaptic plasticity but may also prevent the broader network shifts typically driven by chronic glucocorticoid exposure. By potentially preventing stress-induced hyperactivity in GABAergic interneurons—which would otherwise heavily suppress pyramidal cell output—MR signaling in these cells appears to preserve a critical neuroplasticity essential for adaptive responses to chronic stress.

A critical aspect of our findings is the pronounced sex difference in the behavioral consequences of GABAergic MR deletion. Unlike their male counterparts, female MR^Dlx^ mice did not exhibit the same baseline cognitive deficits or subsequent behavioral resistance to chronic social stress. This discrepancy aligns with a vast body of literature demonstrating profound sexual dimorphism in HPA axis reactivity and behavioral adaptations (Heck and Handa, 2019; Hodes et al., 2024). Also in relation to MR function, previous research has shown that following chronic stress, increased MR expression was found only in the hippocampal CA3 region of female, but not male rats, paralleling their spatial memory improvement (Kitraki et al., 2004). Such sexually divergent molecular adaptations suggest that the baseline excitatory-inhibitory balance, or the specific regulatory role of MR within female limbic networks, may be differentially modulated, potentially in relation to circulating gonadal hormones. Consequently, the loss of MR in GABAergic neurons likely intersects with distinct baseline network states in females, underscoring the necessity of treating sex as a critical biological variable in the neurobiology of stress (Diester et al., 2019).

Despite these novel insights, several limitations of the present study must be acknowledged. First, while the Dlx-Cre driver line successfully restricted MR deletion to inhibitory networks, it induces recombination in all forebrain GABAergic interneurons. Given that distinct interneuron subpopulations, such as parvalbumin and somatostatin-expressing cells, innervate different subcellular compartments of pyramidal neurons and orchestrate distinct behavioral adaptations (Qi et al., 2025), future studies utilizing more granular, subtype-specific Cre lines are required to pinpoint the exact cellular locus of these MR-dependent effects. As MR deletion in our model starts very early in prenatal development, it is also possible that the observed behavioral and physiological differences may be caused by altered developmental processes rather than the acute effects of MR signaling. Second, the behavioral phenotyping of the female mice was less extensive than that of the males and was not conducted in parallel with the male cohorts. Because of this, direct statistical comparisons between the sexes and definitive mechanistic conclusions regarding the female MR^Dlx^ phenotype are limited. Future investigations should incorporate parallel behavioral assessments and *in vitro* electrophysiological recordings in both sexes to fully elucidate the synaptic and circuit-level basis of these sexually dimorphic stress responses.

In conclusion, the present study demonstrates that MRs within GABAergic interneurons function as critical gatekeepers of stress-induced cognitive impairment and synaptic plasticity. Unlike their glutamatergic counterparts, which primarily regulate basal emotional tone and anxiety, GABAergic MRs act to constrain excitatory networks during aversive challenges. Consequently, their ablation confers profound behavioral and synaptic resilience to both acute and chronic stress in male mice. Ultimately, these findings underscore that the cell-type-specific balance of corticosteroid signaling within limbic circuits define stress adaptation and are crucial for our understanding of stress vulnerability and resilience.

## Funding

Huanqing Yang was supported by the Chinese Scholarship Council.

## CRediT authorship contribution statement

**Huanqing Yang:** Formal analysis, Investigation, Visualization, Writing – original draft. **Veronika Kovarova:** Investigation, Writing – review & editing. **Alena Godunova:** Investigation. **Sowmya Narayan:** Investigation. **Lotte van Doeselaar:** Investigation. **Danusa Menegaz:** Investigation. **Matthias Eder:** Investigation, Writing – review & editing. **Juan Pablo Lopez:** Conceptualization, Methodology, Writing – review & editing. **Jan M. Deussing:** Resources, Writing – review & editing. **Mathias V. Schmidt:** Conceptualization, Funding acquisition, Project administration, Resources, Supervision, Writing – review & editing.

## Declaration of competing interest

Given his role as editor in chief of Neurobiology of Stress, Mathias V. Schmidt had no involvement in the peer review of this article and had no access to information regarding its peer review. Full responsibility for the editorial process for this article was delegated to another journal editor.

## Data Availability

Data will be made available on request.

## References

[bib2] Berger S., Wolfer D.P., Selbach O., Alter H., Erdmann G., Reichardt H.M., Chepkova A.N., Welzl H., Haas H.L., Lipp H.-P., Schütz G. (2006). Loss of the limbic mineralocorticoid receptor impairs behavioral plasticity. Proc. Natl. Acad. Sci. U. S. A..

[bib1] Berger S., Wolfer D.P., Selbach O., Alter H., Erdmann G., Reichardt H.M., Chepkova A.N., Welzl H., Haas H.L., Lipp H.P., Schütz G. (2006). Loss of the limbic mineralocorticoid receptor impairs behavioral plasticity. Proc. Natl. Acad. Sci. USA.

[bib3] Bordes J., Miranda L., Reinhardt M., Narayan S., Hartmann J., Newman E.L., Brix L.M., van Doeselaar L., Engelhardt C., Dillmann L., Mitra S., Ressler K.J., Pütz B., Agakov F., Müller-Myhsok B., Schmidt M.V. (2023). Automatically annotated motion tracking identifies a distinct social behavioral profile following chronic social defeat stress. Nat. Commun..

[bib4] Brinks V., Berger S., Gass P., de Kloet E.R., Oitzl M.S. (2009). Mineralocorticoid receptors in control of emotional arousal and fear memory. Horm. Behav..

[bib5] De Donno C., Lopez J.P., Luecken M.D., Kos A., Brivio E., Bordes J., Yang H., Deussing J.M., Schmidt M.V., Theis F.J., Chen A. (2026). Single-cell characterization of the adult male hippocampus suggests a prominent, and cell-type specific, role for Nrgn and Sgk1 in response to a social stressor. Mol. Psychiatr..

[bib6] de Kloet E.R. (2022). Brain mineralocorticoid and glucocorticoid receptor balance in neuroendocrine regulation and stress-related psychiatric etiopathologies. Curr. Opin. Endocr. Metab. Res..

[bib7] De Kloet E.R., Joels M., Holsboer F. (2005). Stress and the brain: from adaptation to disease. Nat Rev.Neurosciences.

[bib8] Diamond D.M., Rose G.M. (1994). Stress impairs LTP and hippocampal-dependent memory. Ann. N. Y. Acad. Sci..

[bib9] Dias B.G., Ressler K.J. (2014). Parental olfactory experience influences behavior and neural structure in subsequent generations. Nat. Neurosci..

[bib10] Diester C.M., Banks M.L., Neigh G.N., Negus S.S. (2019). Experimental design and analysis for consideration of sex as a biological variable. Neuropsychopharmacol.

[bib11] Ennaceur A. (2010). One-trial object recognition in rats and mice: methodological and theoretical issues. Behav. Brain Res..

[bib12] Fluttert M., Dalm S., Oitzl M.S. (2000). A refined method for sequential blood sampling by tail incision in rats. Lab. Anim..

[bib13] Golden S.A., Covington H.E., Berton O., Russo S.J. (2011). A standardized protocol for repeated social defeat stress in mice. Nat. Protoc..

[bib14] Hartmann J. (2025). From intracellular sensors to systemic resilience: reframing the biology of stress. Neurobiol. Stress.

[bib15] Heck A.L., Handa R.J. (2019). Sex differences in the hypothalamic–pituitary–adrenal axis' response to stress: an important role for gonadal hormones. Neuropsychopharmacol.

[bib16] Herman J.P., McKlveen J.M., Ghosal S., Kopp B., Wulsin A., Makinson R., Scheimann J., Myers B. (2016). Regulation of the hypothalamic-pituitary-adrenocortical stress response. Compr. Physiol..

[bib17] Hodes G.E., Bangasser D., Sotiropoulos I., Kokras N., Dalla C. (2024). Sex differences in stress response: classical mechanisms and beyond. Curr. Neuropharmacol..

[bib18] Kanatsou S., Kuil L.E., Arp M., Oitzl M.S., Harris A.P., Seckl J.R., Krugers H.J., Joels M. (2015). Overexpression of mineralocorticoid receptors does not affect memory and anxiety-like behavior in female mice. Front. Behav. Neurosci..

[bib19] Kanatsou S., Ter Horst J.P., Harris A.P., Seckl J.R., Krugers H.J., Joëls M. (2015). Effects of mineralocorticoid receptor overexpression on anxiety and memory after early life stress in female mice. Front. Behav. Neurosci..

[bib20] Kitraki E., Kremmyda O., Youlatos D., Alexis M.N., Kittas C. (2004). Gender-dependent alterations in corticosteroid receptor status and spatial performance following 21 days of restraint stress. Neuroscience.

[bib21] Kupcova I., Danisovic L., Grgac I., Harsanyi S. (2022). Anxiety and depression: what do we know of neuropeptides?. Behav. Sci..

[bib22] Lach G., de Lima T.C.M. (2013). Role of NPY Y1 receptor on acquisition, consolidation and extinction on contextual fear conditioning: dissociation between anxiety, locomotion and non-emotional memory behavior. Neurobiol. Learn. Mem..

[bib23] Lai M., Horsburgh K., Bae S.E., Carter R.N., Stenvers D.J., Fowler J.H., Yau J.L., Gomez-Sanchez C.E., Holmes M.C., Kenyon C.J., Seckl J.R., Macleod M.R. (2007). Forebrain mineralocorticoid receptor overexpression enhances memory, reduces anxiety and attenuates neuronal loss in cerebral ischaemia. Eur. J. Neurosci..

[bib24] Mineur Y.S., Prasol D.J., Belzung C., Crusio W.E. (2003). Agonistic behavior and unpredictable chronic mild stress in mice. Behav. Genet..

[bib25] Monory K., Massa F., EgertovÇ M., Eder M., Blaudzun H., Westenbroek R., Kelsch W., Jacob W., Marsch R., Ekker M., Long J., Rubenstein J.L., Goebbels S., Nave K.A., During M., Klugmann M., Wçôlfel B., Dodt H.U., ZieglgÇÏnsberger W., Wotjak C.T., Mackie K., Elphick M.R., Marsicano G., Lutz B. (2006). The endocannabinoid system controls key epileptogenic circuits in the hippocampus. Neuron.

[bib26] Paré W.P., Glavin G.B. (1986). Restraint stress in biomedical research: a review. Neurosci. Biobehav. Rev..

[bib27] Paul S.N., Wingenfeld K., Otte C., Meijer O.C. (2022). Brain mineralocorticoid receptor in health and disease: from molecular signalling to cognitive and emotional function. Br. J. Pharmacol..

[bib28] Pavlides C., Watanabe Y., McEwen B.S. (1993). Effects of glucocorticoids on hippocampal long-term potentiation. Hippocampus.

[bib29] Qi C., Sima W., Mao H., Hu E., Ge J., Deng M., Chen A., Ye W., Xue Q., Wang W., Chen Q., Wu S. (2025). Anterior cingulate cortex parvalbumin and somatostatin interneurons shape social behavior in male mice. Nat. Commun..

[bib30] Rozeboom A.M., Akil H., Seasholtz A.F. (2007). Mineralocorticoid receptor overexpression in forebrain decreases anxiety-like behavior and alters the stress response in mice. Proc. Natl. Acad. Sci. USA.

[bib31] Sajadi A.A., Samaei S.A., Rashidy-Pour A. (2006). Intra-hippocampal microinjections of anisomycin did not block glucocorticoid-induced impairment of memory retrieval in rats: an evidence for non-genomic effects of glucocorticoids. Behav. Brain Res..

[bib32] Schmidt M.V., Sterlemann V., Ganea K., Liebl C., Alam S., Harbich D., Greetfeld M., Uhr M., Holsboer F., Müller M.B. (2007). Persistent neuroendocrine and behavioral effects of a novel, etiologically relevant mouse paradigm for chronic social stress during adolescence. Psychoneuroendocrinology.

[bib33] Scott K.A., Eikenberry S.A., Elsaafien K., Baumer-Harrison C., Johnson D.N., Sá J.M., Bartolomucci A., Sumners C., Krause E.G., de Kloet A.D. (2025). Cardiometabolic and anxiogenic consequences of chronic social defeat stress in male mice. Neurobiol. Stress.

[bib34] Smythe J.W., Murphy D., Timothy C., Costall B. (1997). Hippocampal mineralocorticoid, but not glucocorticoid, receptors modulate anxiety-like behavior in rats. Pharmacol. Biochem. Behav..

[bib35] Sterlemann V., Ganea K., Liebl C., Harbich D., Alam S., Holsboer F., Müller M.B., Schmidt M.V. (2008). Long-term behavioral and neuroendocrine alterations following chronic social stress in mice: implications for stress-related disorders. Horm. Behav..

[bib36] ter Horst J.P., van der Mark M.H., Arp M., Berger S., de Kloet E.R., Oitzl M.S. (2012). Stress or no stress: mineralocorticoid receptors in the forebrain regulate behavioral adaptation. Neurobiol. Learn. Mem..

[bib38] van Doeselaar L., Yang H., Bordes J., Brix L., Engelhardt C., Tang F., Schmidt M.V. (2020). Chronic social defeat stress in female mice leads to sex-specific behavioral and neuroendocrine effects. Stress.

[bib37] van Doeselaar L., Yang H., Bordes J., Brix L., Engelhardt C., Tang F., Schmidt M.V. (2021). Chronic social defeat stress in female mice leads to sex-specific behavioral and neuroendocrine effects. Stress.

[bib39] Wagner K.V., Marinescu D., Hartmann J., Wang X.-D., Labermaier C., Scharf S.H., Liebl C., Uhr M., Holsboer F., Müller M.B., Schmidt M.V. (2012). Differences in FKBP51 regulation following chronic social defeat stress correlate with individual stress sensitivity: influence of paroxetine treatment. Neuropsychopharmacology.

[bib40] Wagner K.V., Wang X.-D., Liebl C., Scharf S.H., Müller M.B., Schmidt M.V. (2011). Pituitary glucocorticoid receptor deletion reduces vulnerability to chronic stress. Psychoneuroendocrinology.

[bib41] Yang H., Narayan S., Bordes J., van Doeselaar L., De Donno C., Eder M., Menegaz D., Huettl R.-E., Brix L.-M., Mitra S., Springer M., Müller M.B., Chen A., Deussing J.M., Lopez J.P., Schmidt M.V. (2026). Mineralocorticoid receptor in glutamatergic neurons modulates anxiety exclusively in Male mice via regulation of the actin-bundling factor Fam107a. Biol. Psychiatr. Glob. Open Sci..

[bib42] Yang H., Narayan S., Schmidt M.V. (2023). From ligands to behavioral outcomes: understanding the role of mineralocorticoid receptors in brain function. Stress.

[bib43] Zhou M., Bakker E.H.M., Velzing E.H., Berger S., Oitzl M., Joëls M., Krugers H.J. (2010). Both mineralocorticoid and glucocorticoid receptors regulate emotional memory in mice. Neurobiol. Learn. Mem..

[bib44] Zhou M., Kindt M., Joëls M., Krugers H.J. (2011). Blocking mineralocorticoid receptors prior to retrieval reduces contextual fear memory in mice. PLoS One.

